# Antimicrobial Evaluation of Sequentially extracted Leaf of *Vernonia auriculifera* Hiern (*Rejicho*)

**DOI:** 10.1186/s12906-022-03690-2

**Published:** 2022-08-12

**Authors:** Teshale Etiso Wado, Sultan Suleman, Tesfaye Mohammed

**Affiliations:** 1 Pharmacy Department, Arba Minch University, Arba Minch, Ethiopia; 2 School of Pharmacy, Jimma University, Jimma, Ethiopia; 3 Jimma University Laboratory of Drug Quality (JuLaDQ), Jimma University, Jimma, Ethiopia

**Keywords:** Traditional medicine, Vernonia auriculifera, Antibacterial, Antifungal, Phenol, Flavonoid

## Abstract

**Background:**

Though there are reports about *V. auriculifera* antimicrobial activity, there is not enough information about its activity on some bacterial and fungal species. Besides, there was no quantitative evaluation done for phytochemicals previously. Therefore, the main purpose of this research work is to evaluate the antimicrobial activity and quantitative evaluation of the phenol and flavonoid contents of *V. auriculifera*.

**Objectives:**

The objective of this study was to conduct the antimicrobial and quantitative evaluation of a sequentially extracted leaf of *Vernonia auriculifera Hiern*.

**Methods:**

The leaves of the plant were cleaned with tap water and air-dried. The grounded leaf product was subsequently extracted by hexane, chloroform, and methanol in maceration flasks. Then the total phenol and total flavonoid content in each extract were determined. Standard strains of bacterial and fungal species were used to assess the antibacterial, and antifungal susceptibility test and to determine the minimum inhibitory concentration of crude extract.

**Result:**

Extraction yield for hexane, chloroform, and methanol was 0.6 ± 0.05%, 1.7 ± 0.02%, and 3.3 ± 0.01% respectively. The total phenolic content of methanol extract was 72.998 ± 0.002 mg GAE /g. The total flavonoid content of hexane and chloroform extracts were 2.59 ± 0.004 mg QE/g and 9.6 ± 0.02 mg QE/g respectively. The antimicrobial activity test showed the chloroform extract was the most active against all test microorganisms.

**Conclusion:**

This study has shown the activity of *V. auriculifera* against selected microorganisms of study. The chloroform extract was the most active as compared to the hexane and methanol extracts.

**Supplementary Information:**

The online version contains supplementary material available at 10.1186/s12906-022-03690-2.

## Introduction

About 60–85% of the population in every country in the developing world relies on traditional medicine [1, 2]. In Africa, up to 80% of the population uses traditional medicine for primary health care [3].

The WHO report makes a clear case that resistance to the anti-bacterial drug has reached a significant level in many parts of the world indicating that many of the currently available treatment options for common infections are becoming ineffective [4]. Besides advocating the proper utilization of antibiotics [5], focusing on and funding research based on indigenous knowledge of traditional medicines is crucial to come up with novel antimicrobials agents that may serve as newer antibiotics [6].

Plant materials are a crucial part of traditional medicine and are used over-the-counter throughout the world as home remedies [7]. Among plants used for their medicinal value, different species of *Vernonia* are well known worldwide. *Vernonia* is the largest genera belonging to the *Vernonieae* tribe, one of the thirteen tribes that constitute the *Asteraceae* family which represents one of the largest families of flowering plants [8]. *Vernonia auriculifera* is an indigenous to Ethiopia which is either a shrub or small tree that can grow up to 6 m high. Its stem is densely branched, branches striate, lenticellate, glabrous to hairy tomentose in terminal parts [9]. It is widely distributed in the southwestern and southern parts of Ethiopia; it is found in humid lowland woodland, wooded grassland, and scrub, on rocky slopes and along forest edges, at altitudes between 1600 and 2800 m [10].

Though there are reports about *V. auriculifera* antimicrobial activity, there is not enough information about its activity on some bacterial and fungal species [11–20]. Besides, there was no quantitative evaluation previously done for its crude extract. Therefore, the main purpose of this research work is to evaluate the antimicrobial activity and quantitative evaluation of the phenol and flavonoid contents *V. auriculifera.*

## Objective

### General Objective

**Fig. 1:**
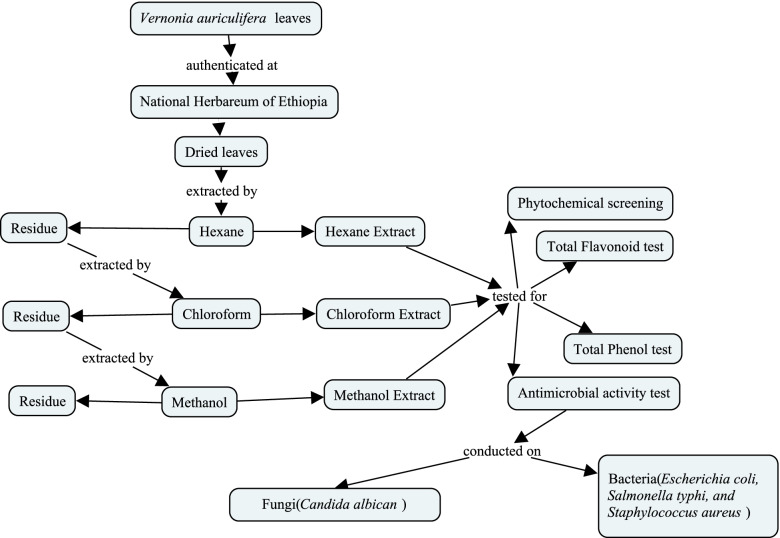
Flowchart of sequential extraction and antimicrobial testing of *V. auriculifera* [21]

The objective of this study was to conduct an Antimicrobial Evaluation of Sequentially extracted leaves of *Vernonia auriculifera* Hiern (Rejicho) (Figure 1).

### Specific Objective

- To quantify two phytochemicals of *V*. *auriculifera* extracts.
- To evaluate the antimicrobial property of *V. auriculifera*.

## Materials and methods

### Materials, chemicals, and reagents

#### Instruments and apparatus

Specord 200 plus UV spectroscopy (Analytic Jena, Germany), 30 W Vortex Mixer (Xh-D Continuous Working, China), Muffle Furnace (Uma Instruments, India), Elmasonic ultrasonic devices (Imlab, Germany), stirring hot plate (Cimarec, USA), WT50001NF Electronic balance (WANT Balance Instrument Co. Ltd, China), WB01water bath (India Mart, India), rotary evaporator (Heidolph, Germany), Oven (Genlab Ltd, UK), maceration flask, test tubes, distillation flask, beakers, and funnels are instruments and apparatus used to do this research.

#### Chemicals and reagents

Chloroform 99.6% (analytical grade, Trust Chemical Laboratories), hexane 85% (analytical grade, Loba Chemie PVT.LTD), ethyl acetate 99.8% (analytical grade, Loba Chemie PVT.LTD), methanol 99% (analytical grade, Abron Exports- 133,001 India) and Mueller Hilton agar media (HiMedia, India) is used as culture media procured from Merry Chemicals Supplier, Addis Ababa. Hydrochloric acid, Wagner’s reagent, sodium hydroxide, powder ferric chloride, acetic anhydride, and sulfuric acid reagents were donated by Jimma University Pharmaceutical Quality Control Laboratory. And Gallic acid standard was obtained from the Jimma University Chemistry department.

### Plant collection, identification, and preparation

The leaves of *Vernonia auriculifera* Hiern were collected from the Sidama region, for easy identification of the species from *Vernonia myrantha*. The collection of this medicinal plant was done in the morning to imitate the collection procedure used by local traditional healers. The plant identification was performed at the National Herbarium of Addis Ababa University by Melaku Wendafrash, senior botanist, and a voucher specimen was deposited with the TE001 voucher number. The leaves of the plant were washed thoroughly with running tap water and the plant sample was air-dried at room temperature under a shade, and ground into a coarse powder using a pestle and mortar. The coarsely powdered plant material was packed in plastic bags until extraction [7].

### Determination of extractable matter

To a conical flask, 4.0 g of the coarsely powdered air-dried leaf was placed and 100 ml of water was added and weighed. After shaking well it was allowed to stand for 1 h. It was boiled for 1 h, cooled, and weighed. The amount of water lost by evaporation was replaced by readjusting to the original total weight. It was filtered by dry filter paper after shaking well and 25 ml of the filtrate was taken and evaporated to dryness in water bath. Then it was dried in an oven at 105 °C for 6 h and cooled in a desiccator for 30 min, and then it was weighed. The content of extractable matter was calculated in mg per g of air-dried material [7].

The 4.0 g of coarsely powdered leaf material was placed in five conical flasks and macerated with 100 ml of chloroform, ethanol, ethyl acetate, hexane, and methanol for 6 h with frequent shaking and allowed to stand for 18 h. The extracts were filtered and from each 25 ml filtrate was taken to eventually be evaporated to dryness on a water bath. Each extract was dried at 105 °C for 6 h, cooled in a desiccator for 30 min, and weighed [7].

The extractive value of all solvents was calculated using the following equation below [22]:

$$\mathrm{Total\;extract\;yield},\mathrm{ Y }\left(\mathrm{\%}\right)=\frac{\mathrm{The\;total\;mass\;of\;extract }}{Total\;mass\;of\;sample} \times 100$$

### Extraction

**Fig. 2:**
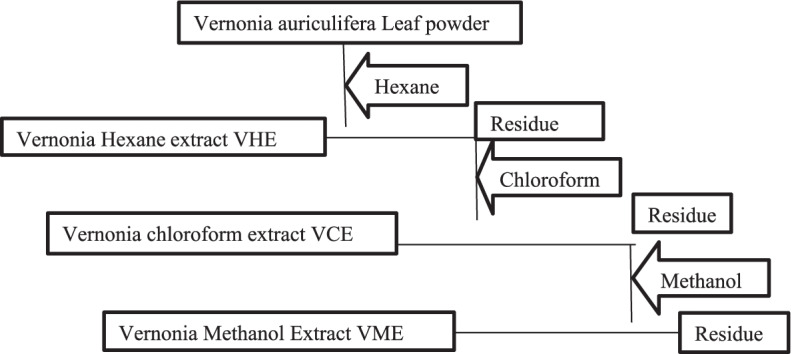
Subsequent extraction of *V. auriculifera*

The coarsely powdered leaf part of the plant was weighed using sensitive balance and was sequentially extracted first in hexane in maceration flasks for 72 h with regular shacking. Then, the extracts were filtered through filter paper. And it was concentrated at 40 °C with a rotary evaporator in a distillation flask to eliminate solvent from the crude extract and concentrated further to dryness in a water bath at 40 °C. Then, the residue was macerated in chloroform, filtered, and concentrated in the same way for hexane extraction. And the residue was extracted by methanol in the same manner. Then, the extracts were stored in tightly closed bottle containers in a refrigerator at 4 °C until they were used for the experiment [23] (Figure 2).

### Phytochemical screening

#### Test for Alkaloids (Wagner’s reagent)

A fraction of crude extract was treated with 3drops of Wagner’s reagent [2 g of potassium iodide and 1.27 g of iodine in 100 mL of water] and observed for the formation of a reddish-brown precipitate (or colorations) [24].

#### Test for Flavonoids (Alkaline reagent test)

2 mL of leaf extracts were separately treated with a few drops of 20% sodium hydroxide solution and observed for the formation of intense yellow color, which turns colorless on the addition of dilute hydrochloric acid [7].

#### Test for Phenols and Tannins (Ferric chloride test)

A fraction of leaf extracts were separately treated with aqueous 5% ferric chloride and observed for the formation of deep blue or black color [24].

#### Test for Saponins (Foam test)

2 mL of leaf extracts were separately added to 6 mL of water in a test tube. The mixture was shaken vigorously and observed for the formation of persistent foam [7].

#### Test for Sterols (Liebermann-Burchard test)

1 mL of leaf extracts was treated with drops of chloroform, acetic anhydride, and concentrated H2SO4 and was observed for the formation of dark pink or red color [25].

#### Test for Terpenoids (Salkowki’s test)

1 mL of chloroform was added to 2 mL of extract followed by the addition of concentrated sulphuric acid. A reddish-brown precipitate produced immediately indicates the presence of terpenoids [7].

#### Test for Quinones

A small portion of each extract was treated with concentrated HCl and was observed for the formation of a yellow precipitate (Or coloration) [7].

### Total phenolic content

The Gallic acid standard was prepared in seven concentration levels (10 µg/ml, 50 µg/ml, 100 µg/ml, 150 µg/ml, 200 µg/ml, 250 µg/ml, and 300 µg/ml) and to prepare the blank 0.5 ml methanol, 2.5 ml 10% Folin-Ciocalteu’s reagent dissolved in distilled water and 4 ml of 7.5% of NaCO3. The 0.1 gm of hexane, chloroform, and methanol extracts were weighed and dissolved in 100 ml of 99% methanol to make the solution with a concentration of 1 mg/ml of each extract. From each solution prepared, 0.5 ml was taken and put into test tubes. To each test tube 2 ml of 10%, Folin-Ciocalteu’s reagent was added and vortexed for 15 s. And after 3 min 4 ml of 7.5% of NaCO3 was added and incubated at room temperature for 30 min until there was color development. The samples were prepared in replicates and the mean value of absorbance was taken. The calibration line was constructed using the Gallic acid standard reading previously prepared in the same manner. The absorbance was measured using a double beam UV spectroscope on the wavelength of 765 nm. The concentration of phenolics was read based on the measured absorbance, (mg/ml) from the calibration line; then it was expressed in terms of Gallic acid equivalent (mg of GA/g of extract) [26]. The total phenolic contents of the extracts were calculated using the equation: $TPC=\frac{CxV}{M}$, where C is the sample concentration from the calibration curve (mg/ml), V is the volume (ml) of the extract, and M represents the weight (g) of the extract used [27].

### Total Flavonoid content

The aluminum chloride colorimetric method has used the determination of the total flavonoid content of the sample. Stock quercetin solution was prepared by dissolving 5.0 mg quercetin in 1.0 mL methanol, then the standard solutions of quercetin were prepared by serial dilutions using methanol (5–80 μg/mL). About 20 mg of each extract was dissolved in 5 mL methanol and sonicated for 45 min. An amount of 0.5 mL diluted standard quercetin solutions or extracts was separately mixed with 0.15 mL of 5% sodium nitrite and incubated for 5 min. Then 0.15 of 10% aluminum chloride was added and incubated for 5 min followed by the addition of 4% sodium hydroxide and the volumes were made up to 5 ml with distilled water. The distilled water was used as a blank. After incubating for 15 min the absorbance of the reaction mixtures was measured against blank at 510 nm wavelength with a UV–Vis spectrophotometer. The concentration of total flavonoid content in the test samples was calculated from the calibration plot and expressed as mg quercetin equivalent (QE)/g of extracts. All the determinations were carried out in triplicate [28]. The total flavonoids contents of the extracts were calculated using the equation: $TFC=\frac{CxV}{M}$, where C is the sample concentration from the calibration curve (mg/ml), V is the volume (ml) of the extract, and M represents the weight (g) of the extract used [27].

### Biological evaluation

#### Test microorganisms and antimicrobial standards

All test microorganisms used for antimicrobial activity tests were obtained from Jimma University, Department of Biology. Pure cultures of bacteria: *E.coli* (ATCC25922), *S. aureus* (ATCC25923), and *S. typhi* (ATCC13311) were maintained on nutrient agar slants at 4 °C, while the fungi: *C. albicans* (ATCC10231) was maintained on Sabourauds’ Dextrose Agar (SDA) at 4 °C. Then they were activated by incubating for 24 h at 37 °C [29]. Chloramphenicol, ciprofloxacin, miconazole, and dimethyl sulfoxide were obtained from the Microbiology department.

#### Antibacterial test

Bacterial cell suspension with an equilibrated concentration of a 0.5 McFarland standard in sterile saline was used to achieve the concentration of 10^7^ CFU/ml. Then the microorganisms were inoculated onto the Mueller Hinton Agar plates. The dissolution of crude extracts and standard antibiotics was done by dimethyl-sulfoxide (DMSO 4%, v/v). Antibacterial activity of the crude extract was tested on activated gram-positive bacteria (*S. aureus*) and gram-negative bacteria (*S. typhi, E. coli*) [30]. The discs of 6 mm diameter were loaded with three plant extracts of a concentration of 500 mg/ml. The positive controls were also loaded on the disks (CAF 62.5 mg/ml for *S. typhi*; Ciprofloxacin 83 mg/ml for *S. aureus* and CAF 62.5 mg/ml for *E. coli*) and dimethyl-sulfoxide was used as the negative control. The three extracts and positive control were placed far from each other to avoid overlap of the zone of inhibition. The cultures were then incubated for 24 h at 37 °C. After incubation, the zone of inhibition of plant extracts was measured and recorded [31].

#### Antifungal test

The *C. albicans* cell suspension of 10^6^ spores/ml was attained by comparing it to 0.5 McFarland. Then it was inoculated onto Sabouraud Dextrose Agar (SDA) plates. The three solvents extract were prepared at a 500 mg/ml concentration level. The disks were immersed in the positive control (Miconazole 20 mg/ml), three extracts, and negative control (DMSO) and placed onto the prepared media. The cultures were incubated for 48 h at 25 °C [31].

#### Determination of minimum inhibitory concentrations (MIC) of the extracts

MIC is defined as the lowest concentration of the antimicrobial agent that inhibits microbial growth after 24 h of incubation. Each extract was manipulated to determine its MIC using the disk diffusion method against the respective microorganisms by different concentration levels. The hexane extract was prepared at 175, 350, and 700 concentration levels. The concentration of chloroform extract was prepared at 43.75, 87.5, 175, 350, and 700 mg/ml. The methanol extract was prepared at 350 and 700 mg/ml concentration levels. The bacterial and fungal strains were inoculated on Mueller-Hilton agar and SDA respectively. The discs were then loaded with different concentrations of the three solvent plant extracts and placed on the top of the agar plates allowing enough spaces between them. The bacterial and fungus cultures were incubated for 24 h at 37 °C and 48 h at 25 °C respectively. Then using a ruler the respective zones which had no growth of microorganisms were measured and recorded [32].

#### Determination of minimum bactericidal concentrations (MBC)

A small portion was taken from the microorganism growth-free zone of the two lowest concentrations of each extract which showed growth inhibition in the MIC test and subcultured onto sterile Mueller Hinton Agar plates. Then the plates were incubated at 35 ^0^C for 24 h and examined for bacterial growth corresponding to plant extract concentration. The concentration of plant extracts that did not show any bacterial growth was taken as MBC [32].

### Data analysis and interpretation

The experimental data are expressed as mean ± standard error of the mean (SEM). Microsoft excel 2010 was used to process the data. The phytochemical constituent data was compared against a previously done study. Diameters of zones of inhibition of the extracts were measured (mm) and compared to the control standard. The one-way analysis of variance (ANOVA) was used to compare the mean zone of inhibition of the extract activity against each microorganism at a significance level of *p* < 0.05. The gallic acid calibration standard curve was used to find the concentration of phenolics. The quercetin standard curve was used to get the total flavonoid content in each extract.

### Quality assurance

Standard Operating Procedures (SOPs) were developed and used to ensure reliability for all tests. All quality control and assurance measures were taken including calibration check measures and replicate analysis of samples. Also, clean and dry glassware was used in sample preparation to avoid contamination of the solutions prepared. The laboratory results generated were entered in a logbook for safety. Positive and negative control groups were prepared for an anti-bacterial and anti-fungal test.

### Ethical clearance

The proposal of this research was reviewed by Jimma University Institute of Health, Institutional Review Board. Every laboratory protocol was verified and assured the safety of the investigator and the investigator used a glove, white coat, and safety shoe during the laboratory investigation. The reagents and solvents chosen to be used during this experimental work were handled in an environmentally friendly manner. In communicating with different offices regards to this research, the official paper was sent from the CBE office, Pharmacy department, and principal advisor.

## Results

### Extractive values of different solvents and phytochemical screening

The extractive value of six solvents has shown different values that water had the highest and Hexane had the lowest percentage yield (Table 1).

**Table 1:** Extractive values of different solvents

Solvents	extractive value
Chloroform	1.70 ± 0.02%
Ethanol	5.07 ± 0.25%
Ethyl acetate	1.3 ± 0.6%
Hexane	0.60 ± 0.05%
Methanol	3.30 ± 0.01%
Water	11.02 ± 0.35%

The phytochemical screening showed positive and negative results for the three extracts. There was a reddish-brown precipitate formation after treating chloroform and methanol extracts with Wagner’s reagent. Treating the hexane and chloroform extracts with alkaline reagents showed the presence of flavonoids. The addition of ferric chloride to the extracts of hexane, chloroform, and methanol formed sand yellow, fade green, and deep blue colors respectively. The persistent foam formation was observed only for methanol extract. Sterol test has shown the formation of a dark pink color only for hexane and chloroform extracts. Salkowki’s test has produced reddish-brown precipitate in the test of all three extracts. There was no yellow precipitate or color formation after treating each extract with concentrated hydrochloric acid. A deep green color was observed after treating the chloroform extract with hydrochloric acid (Table 2).

**Table 2:** Phytochemical tests of *V*. *auriculifera* extracted by three solvents

S.N	Tests	n-Hexane extract	Chloroform extract	Methanol extract
1	Alkaloids (Wagner’s reagent test)	-	+	+
2	Flavonoids (Alkaline reagent test)	+	+	-
3	Phenols and Tannins (Ferric chloride test)	-	-	+
4	Saponins (Foam test)	-	-	+
5	Sterols (Liebermann-Burchard test)	+	+	-
7	Terpenoids (Salkowki’s test)	+	+	+
8	Quinones	-	-	-

### Total phenolic content

**Fig. 3:**
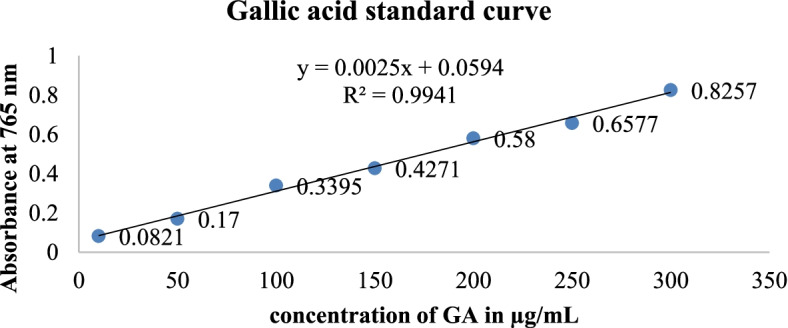
Determination of total phenols

The total phenolic content was evaluated for three extracts and there was the blue coloration of methanol extract after the addition of sodium carbonate and incubation for 30 min. There was no color development observed for hexane and chloroform extracts. Using the formula, $TPC=\frac{CxV}{M}$, the concentration of phenols was calculated to be 72.998 ± 0.002 mg GAE/g for methanol extract while the hexane extract and chloroform extract reading showed no absorbance. (Figure 3).

### Total flavonoid content

**Fig. 4:**
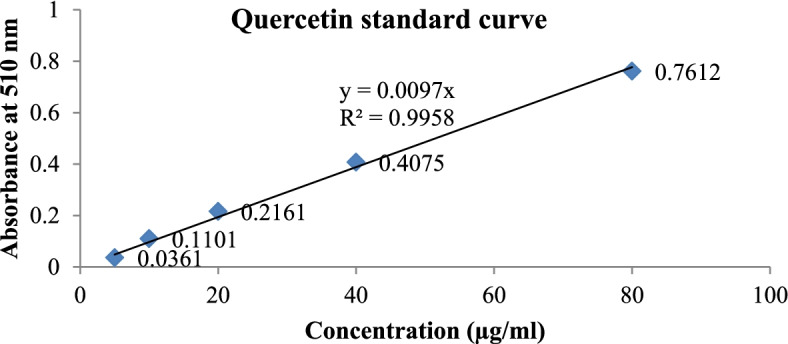
Determination of total flavonoids

The concentration from the graph was used to calculate flavonoid content in each extract by the formula, $=\frac{CxV}{M}$. The chloroform extract gave the highest flavonoid content (9.60 ± 0.02 mg QE/g) followed by the hexane extract (2.59 ± 0.004 mg QE/g). The methanol extract had no absorbance. (Figure 4).

### Antimicrobial activity test

The antimicrobial activity of three extracts was done at a 500 mg/ml concentration level and the chloroform extract was active against all microorganisms in the study as shown in Table 3. There was a significant activity of chloroform extract against *C. albicans* than in the rest of the microorganisms in the study (*p *< 0.05). The hexane had moderate activity against *E. coli* (13 ± 1) at 500 mg/ml. But it has significantly lower activity than chloroform extract’s activity against *E. coli* (*p* < 0.05) at the same concentration level. The methanol extract was inactive against all study microorganisms at 500 mg/ml.

**Table 3:** Zone of inhibitions of three extracts of *V. auriculifera*

The diameter of inhibition (mm)						
	Hexane extract (500 mg/ml)	Chlororform extract (500 mg/ml)	Methanol extract (500 mg/ml)	Ciprofloxacin (83 mg/ml)	Chloramphenicol ( 62.5 mg/ml)	Miconazol (20 mg/ml)
Candida albican	0	23 ± 0.5	0	-	-	12 ± 2
Escherichia coli	13 ± 1	18 ± 1	0	-	28 ± 1	-
Salmonella typhi	0	13 ± 0.7	0	-	31 ± 1	-
Staphylococcus aureus	0	15 ± 0.9	0	26 ± 1	-	-

(-) = not examined

### Minimum Inhibition Concentration and Minimum Bactericidal Concentration

The minimum inhibitory concentration (MIC) of two solvent extracts (hexane and chloroform) was depicted in Table 4. The methanol extract was inactive at both concentration levels of 350 mg/m and 700 mg/ml. The inhibitory effects of hexane extract start at 350 mg/ml with an inhibition zone of 8 mm against *E.coli* but it was less effective against the rest of the microorganisms.

**Table 4:** MIC of three solvent extracts of *V. auriculifera*

Plant extracts	Concentration (mg/ml)	Inhibition Zone (mm)			
		S. typhi	E. coli	S.aureous	C.albican
Hexane	175	0 ± 0	0 ± 0	0 ± 0	0 ± 0
	350	0 ± 0	8 ± 2	0 ± 0	0 ± 0
	700	12 ± 0.5	20 ± 1.5	9 ± 0.3	12 ± 0.6
Chloroform	43.75	0 ± 0	0 ± 0	0 ± 0	0 ± 0
	87.5	0 ± 0	0 ± 0	0 ± 0	10 ± 2
	175	0 ± 0	0 ± 0	8 ± 0.7	13 ± 0.8
	350	12 ± 1.8	13 ± 1.4	15 ± 2	22 ± 2.3
	700	25 ± 3	26 ± 0.6	24 ± 2.5	27 ± 1

The chloroform extract inhibition effect starts at 87.5 mg/ml against *C. albicans* which agrees with the result of the preliminary activity test.

The minimum bacterial concentration (MBC) was confirmed by the absence of bacterial growth of the tested strains streaked from the inhibition zone corresponding to their lowest MIC. The chloroform extract has shown the best activity against *C. albicans* at an MBC of 175 mg/ml while the MBC for *S. typhi*, *E. coli,* and *S. aureus* was 350 mg/ml. The hexane extract had shown to have an activity having a high MBC level at 700 mg/ml.

## Discussion

Traditionally used medicinal plants are an important source of modern medicine as they constitute the most active phytochemicals which are active against multiple human ailments. Out of active compounds isolated from a plant source, more than 121 are currently part of modern medicine [33].

Herbal medications are useful for their low cost, readily available, fewer side effects, and effectiveness for chronic health problems. Besides this, antimicrobial resistance is a great challenge in current medicine [34]. Some traditionally used medicinal plants have essential oils which are effectively active against multiple pathogenic microorganisms including methicillin-resistant bacterial strains [35–37] Despite their use, they do have disadvantages of lack of dosage instruction, and missing scientific proof for their claim [38].

The polarity of extracting solvents significantly affects the yield of medicinal plant extraction [39]. As shown in Table 1 Water had the greatest extractive value as compared to the rest of the solvents and ethanol was the second-highest. Methanol and chloroform were third and fourth in their extractive value. Hexane was used as an initial extractive solvent to remove the fatty/ oily components. Chloroform has medium polarity and extracted the components with medium polarity. Methanol had very high polarity and extracted polar components of the plant. The highest extractive value of water and alcoholic solvents implies *V.auriculifera* has the highest amount of very polar constituents like phenols and tannins [40]. In addition the higher the extractive value of a given solvent implies it has a higher capacity for extracting more bioactive compounds [41].

The phytochemical screening of *V. auriculifera* leaf extract by n-hexane, chloroform, and methanol has shown the presence of alkaloids, saponins, phenolics, flavonoids, sterols, and terpenoids. There were no quinones found in the three extracts. The chloroform extract has shown the greatest number of phytochemical components. These phytochemical components have activity against different pathogenic microorganisms and also have a pharmacological effect on the human body [42]. A study conducted on methanol extract of *V. auriculifera* has shown the plant has flavonoids, tannins, terpenoids, and saponins though it differs from this study that it has shown the absence of alkaloids in methanol extract which could be due to the test method difference [43]. Another study done on the larvicidal activity of different species of vernonia reviled similar results to the current study despite the difference in the method of extraction used [44].

The phenol and flavonoid compounds are known to have multiple biological activities [45, 46]. The extractability of phenolic compounds is highly dependent on the polarity of extracting solvents. Highly polar solvents have a greater capacity to extract these compounds [26]. Total phenolic content analysis revealed that the methanol extract had 72.998 ± 0.002 mg GAE/g. This value was higher than the ethanol extract of *V. amygdaline* which was 63.044 mg GAE/g [47]. The total flavonoid of *V. auriculifera* was 2.59 ± 0.004 mg QE/g and 9.60 ± 0.02 mg QE/g for hexane and chloroform extracts. The total flavonoid content in this study was much lower than that of ethanol extract of *V. amygdaline* which was 94.08 mg QE/g [27].

The antimicrobial activity study of *V. auriculifera* has shown activity against all study microorganisms. But the most active extract was the chloroform extract and there was little activity of hexane extract against *E. coli* bacteria. The methanol extract had no activity against any one of the microorganisms in the study. A study done on methanol extract of *V. auriculifera* has shown the activity of the extract against clinical isolate of *S. aureus* at the concentration level of 200 mg/ml [43]*.* The difference in result could be due to the method of extraction used in this study which is sequential extraction that 200 mg/ml concentration of chloroform activity and methanol activity gave a similar result. Chloroform has a relative polarity of 0.259 which is less than that of methanol which has 0.762 polarities [48]. Thus chloroform have dissolved most of the active phytochemicals, which have medium polarity, rendering the methanol extract inactive [48]. The phytochemical screening result showed there is no sterol in the methanolic extract which is one of the possible reasons for its loss of activity. Sterols are an important terpene subclass that is responsible for multiple pharmacological activities [49–51].

*S. aureus* is one of bacterial species which cause would infection [52]. The activity of *V. auriculifera* against *S. aureus* proves its used to treat a wound in some parts of Ethiopia [17]. *E. coli* and *S.typhi* are known causative agents for diarrhea and thus the activity of chloroform extract is evidence for the claim of this study plant is used to treat diarrheal conditions [53]. Its topical use to treat skin disorders could be due to its significant activity against fungal infection [18].

The MIC and MBC showed that there is much difference between the activities of different solvent extracts. The methanol extract was inactive even at the highest concentration of 700 mg/ml. *C. albicans* was most susceptible relatively to chloroform extract at an 87.5 mg/ml concentration level as compared to the rest of the study microorganisms at a *p* < 0.05. The hexane extract had activity against *E.coli.* A similar result was found with the hexane fraction of *Vernonia cinerea* extract having the greatest activity against *E.coli* [54]*.*

This study was done using a limited number of microorganisms and antibiotics which are the limitations of the study.

## Conclusion

This study has shown that *V. auriculifera* has different types of phytochemicals which are important for plant extract’s biological activities. The crude plant extract is active against selected microorganisms of study. Especially the chloroform extract is the most active as compared to the hexane and methanol extracts. These findings serve as proof of this plant’s traditional utilization.

## Supplementary Information

**Additional file 1.**

## Data Availability

The datasets used and/or analyzed during the current study are available from the corresponding author on reasonable request.

## Abbreviations

CAF: Chloramphenicol
DMSO: Dimethyl sulfoxide
EtOAc: Ethyl acetate
H_2_SO_4_: Sulfuric acid
HCL: Hydrochloric acid
MBC: Minimum bacterial concentration
MIC: Minimum inhibitory concentration
MO: Microorganism
MQL: Method Quantification Limits
NMR: Nuclear magnetic resonance
PVT, LTD: Private limited
SSA: Sub-Saharan Africa
SOP: Standard Operation System
TM: Traditional Medicine
UV: Ultra Violate
WHO: World Health Organization
