# Supplementary material for: Antimicrobial Evaluation of Sequentially extracted Leaf of Vernonia auriculifera Hiern (Rejicho)
Source: BMC Complement Med Ther. 2022 Aug 12;22:219. doi: 10.1186/s12906-022-03690-2 (PMC9373463; doi:10.1186/s12906-022-03690-2)
Supplement: Supplementary file 1 — Additional file 1. [file 12906_2022_3690_MOESM1_ESM.docx]

# Appendix


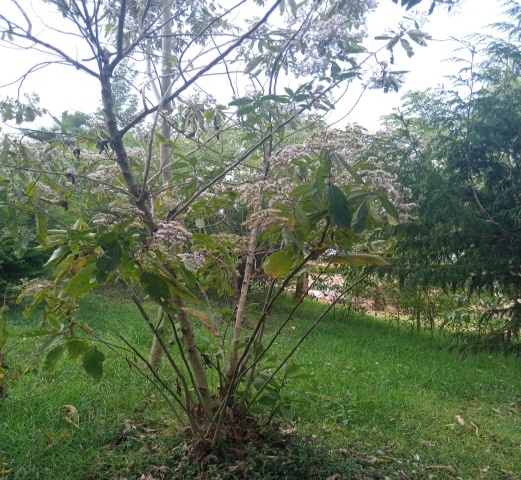


figure S1: the adult shrub of *V. auriculifera*


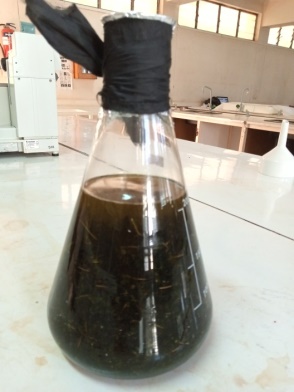


figure S2: Maceration


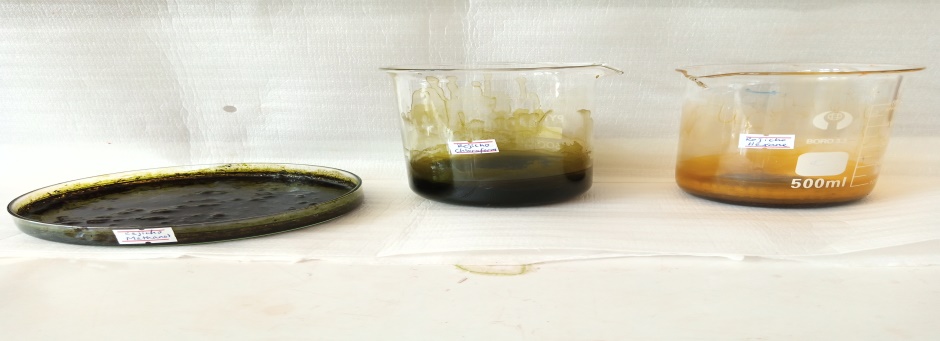


figure S3: Crude extracts


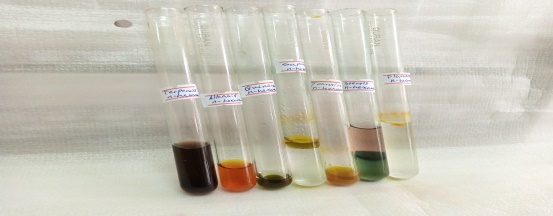

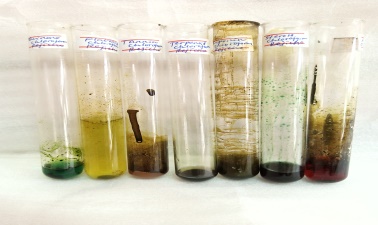

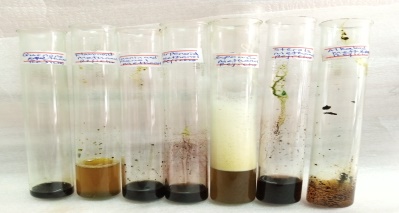


figure S4: phytochemical screening of n-hexane, chloroform and methanol extracts


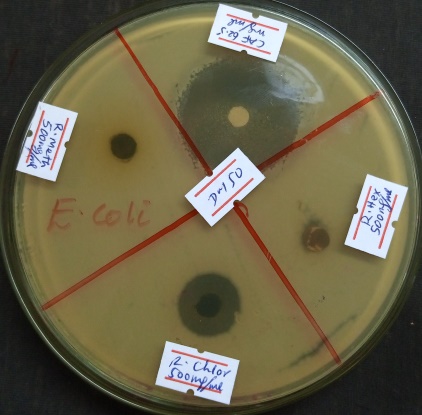

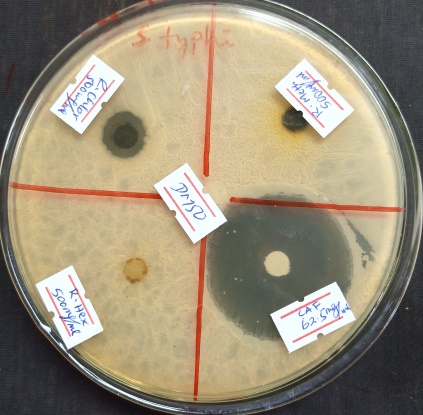


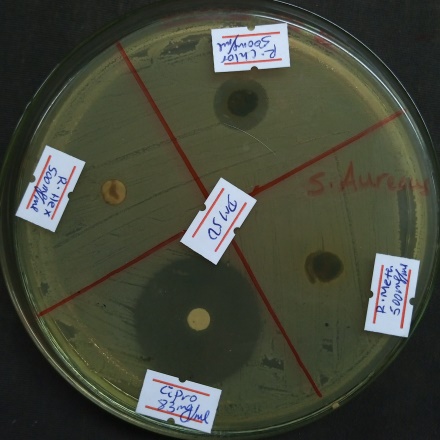

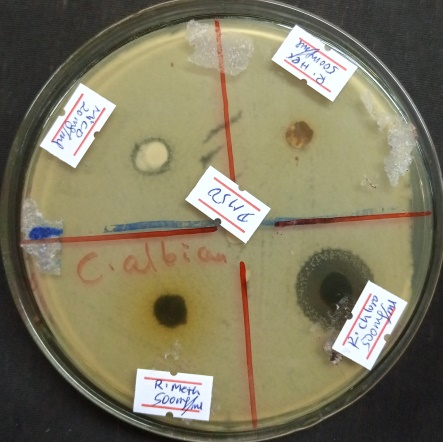


figure S5: pictures of microorganisms’ growth inhibition


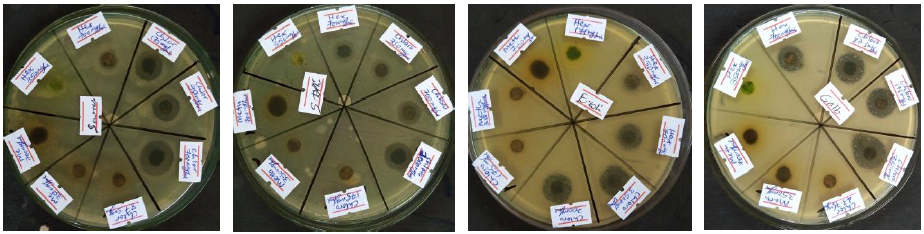


figure S6: Minimum inhibitory concentration of three solvent extracts of *V. auriculifera*
